# Intrinsic Disorder in Protein Interactions: Insights From a Comprehensive Structural Analysis

**DOI:** 10.1371/journal.pcbi.1000316

**Published:** 2009-03-13

**Authors:** Jessica H. Fong, Benjamin A. Shoemaker, Sergiy O. Garbuzynskiy, Michail Y. Lobanov, Oxana V. Galzitskaya, Anna R. Panchenko

**Affiliations:** 1 National Center for Biotechnology Information, National Institutes of Health, Bethesda, Maryland, United States of America; 2 Institute of Protein Research, Russian Academy of Sciences, Pushchino, Moscow Region, Russia; Indiana University-Purdue University, United States of America

## Abstract

We perform a large-scale study of intrinsically disordered regions in proteins and protein complexes using a non-redundant set of hundreds of different protein complexes. In accordance with the conventional view that folding and binding are coupled, in many of our cases the disorder-to-order transition occurs upon complex formation and can be localized to binding interfaces. Moreover, analysis of disorder in protein complexes depicts a significant fraction of intrinsically disordered regions, with up to one third of all residues being disordered. We find that the disorder in homodimers, especially in symmetrical homodimers, is significantly higher than in heterodimers and offer an explanation for this interesting phenomenon. We argue that the mechanisms of regulation of binding specificity through disordered regions in complexes can be as common as for unbound monomeric proteins. The fascinating diversity of roles of disordered regions in various biological processes and protein oligomeric forms shown in our study may be a subject of future endeavors in this area.

## Author Summary

Traditionally, protein structure is believed to determine function. Recently, it was observed that many proteins contain regions without well-defined structure (intrinsically disordered regions), including a large fraction of eukaryotic proteins. Intrinsic disorder has been associated with particular functions including cell regulation; signaling; and protein, DNA, and ligand binding. Many proteins are intrinsically disordered in native form and fold upon binding, following the conventional paradigm. Accordingly, disorder in a protein may facilitate binding to multiple partners. However, in some cases disorder has also been found in the bound state. To gain clearer insight into the functional importance of disorder regions in protein complexes, we perform a large-scale analysis of disorder using protein structures in complex and in unbound forms. We show that disorder in protein complexes is rather common and pinpoint changes that occur upon protein binding at interaction interfaces. By illustrating a variety of functional roles for disorder in specific proteins, we emphasize the versatility and importance of this phenomenon.

## Introduction

Many proteins and protein regions have been shown to be intrinsically disordered under native conditions; namely, they contain no or very little well-defined structure [1]–[6]. Intrinsically disordered proteins (IDPs) have been found in a wide scope of organisms and their disorder content was shown to increase with organism complexity [7]–[11]. Comparative analysis of the functional roles of disordered proteins suggest that they are predominantly located in the cell nucleus; are involved in transcription regulation and cell signaling; and also can be associated with the processes of cell cycle control, endocytosis, replication and biogenesis of cytoskeleton [10],[12].

IDPs have certain properties and functions that distinguish them from proteins with well-defined structures. 1) IDPs have no unique three-dimensional structure in an isolated state but can fold upon binding to their interaction partners [1], [4], [13]–[18]. 2) Conformational changes upon binding in proteins with unstructured regions are much larger than those in structured proteins [1]. 3) The conformations of disordered regions in a protein complex are determined not only by the amino acid sequences but also by the interacting partners [1],[19]. 4) IDPs can have many different functions and can bind to many different partners using the same or different interfaces [20]. 5) IDPs can accommodate larger interfaces on smaller scaffolds compared to proteins with well-defined structure [14],[21],[22]. 6) IDPs typically have an amino acid composition of low aromatic content and high net charge as well as low sequence complexity and high flexibility [2],[10],[23]. 7) Intrinsic disorder provides for a rapid degradation of unfolded proteins, thereby enabling a rapid response to changes in protein concentration (regulation through degradation) [24]. 8) Finally, intrinsic disorder offers an elegant mechanism of regulation through post-translational modifications for many cellular processes [20],[25].

Predictions of disorder in proteins take into account the characteristic features of unstructured proteins and have been shown to be rather successful, especially in the case of large regions. According to the results of CASP7 (7th Community-Wide Experiment on the Critical Assessment of Techniques for Protein Structure Prediction), the best prediction groups successfully identified 50–70% of the disordered residues with false positive rates from 3% to 16% [26]. Prediction methods aim to identify disordered regions through the analysis of amino acid sequences using mainly the physico-chemical properties of the amino acids [23], [27]–[36] or evolutionary conservation [12], [37]–[39].

As protein interactions are crucial for protein function ([40], references within), the biological role of disordered proteins should also be studied in this context. Indeed, folding of disordered proteins into ordered structures may occur upon binding to their specific partners [1], [4], [13]–[17] which may allow disordered regions to structurally accommodate multiple interaction partners with high specificity and low affinity [1], [41]–[43]. Moreover, it has been shown that the binding mechanism, whether binding occurs between folded or unfolded chains, depends on the structural characteristics, interface properties, and degree of minimal frustration of monomers [21],[44]. Binding through unfolded or partially unfolded intermediates can provide a kinetic advantage through the “fly-casting” mechanism [19]. According to this mechanism a dimensionality reduction occurs when the folding of a disordered protein is coupled with binding, thereby speeding up the search for specific targets.

A database of continuous protein fragments (Molecular Recognition Features or MORFs) has been compiled from the Protein Data Bank to include short protein chains (with fewer than 70 residues) bound to larger proteins [45],[46]. It has been argued that MORFs participate in the coupling of binding and folding, a hypothesis that was supported by the analysis of the composition and predicted disorder of MORF segments. As a result of studying the subtle structural differences of the same proteins in different conditions and functional states, many so-called “dual personality” protein segments were found able to exist in both ordered and disordered states [47]. There is a continuous range between completely structured and completely disordered proteins in which intermediate cases are rather common [24]: proteins that are disordered but compact, multi-domain proteins with disordered linkers, and ordered proteins with some local disorder.

Examples of proteins with intrinsically disordered regions which exhibit coupling between folding and binding have been described in the literature previously [1], [4], [13]–[18]. Nevertheless, the universality of this phenomenon and functional importance of many disordered regions remains unclear. The question can be expanded further to how much intrinsic disorder do protein complexes contain and what is its functional importance? To answer these questions we examine observed and predicted disorder in protein complexes and unbound proteins using a large-scale dataset of protein structures. The atomic details of structures and the conserved binding mode analysis introduced earlier [48] allow us to monitor changes happening on or near interaction interfaces and to infer their functional importance.

## Methods

### Assembling the dataset

**Figure 1:**
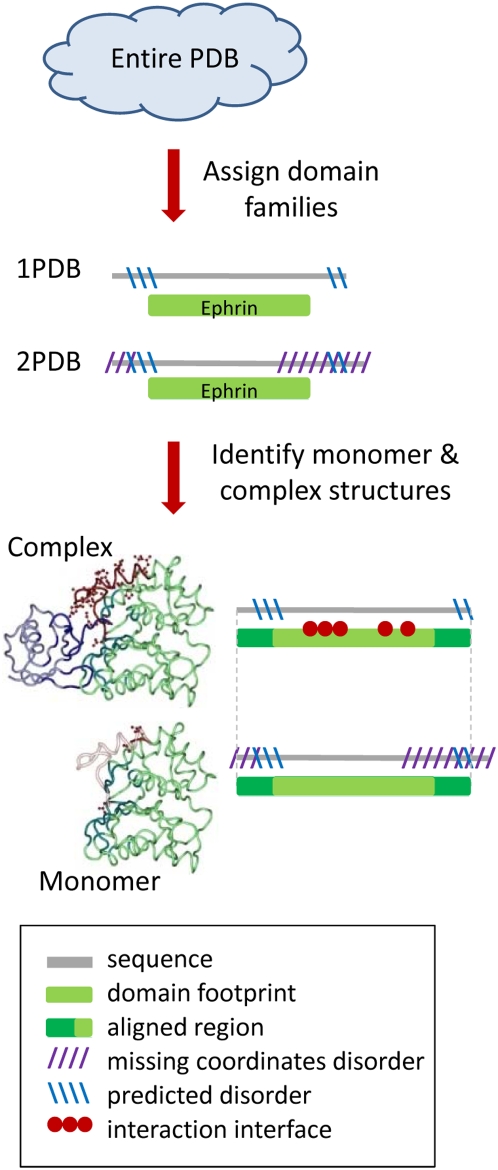
Flowchart showing the construction of the dataset.

Figure 1 presents a flowchart of the assembly of the dataset. From the Protein Data Bank (PDB) [49] we selected X-ray structures with resolution better than 3Å. We assigned domains from the Conserved Domain Database (CDD) [50] on each protein structure chain using RPS-BLAST [51] with default parameters (E-value≤0.01). As we focus on protein-protein interactions (interactions between different protein chains) we ensured that each chain has only one CDD domain which covers at least 70% of the full chain sequence. Among overlapping domain assignments, the domain with the longest footprint was chosen where the footprint region extends from the first to the last residue in the alignment mapping a CDD family to a given chain.

**Figure 2:**
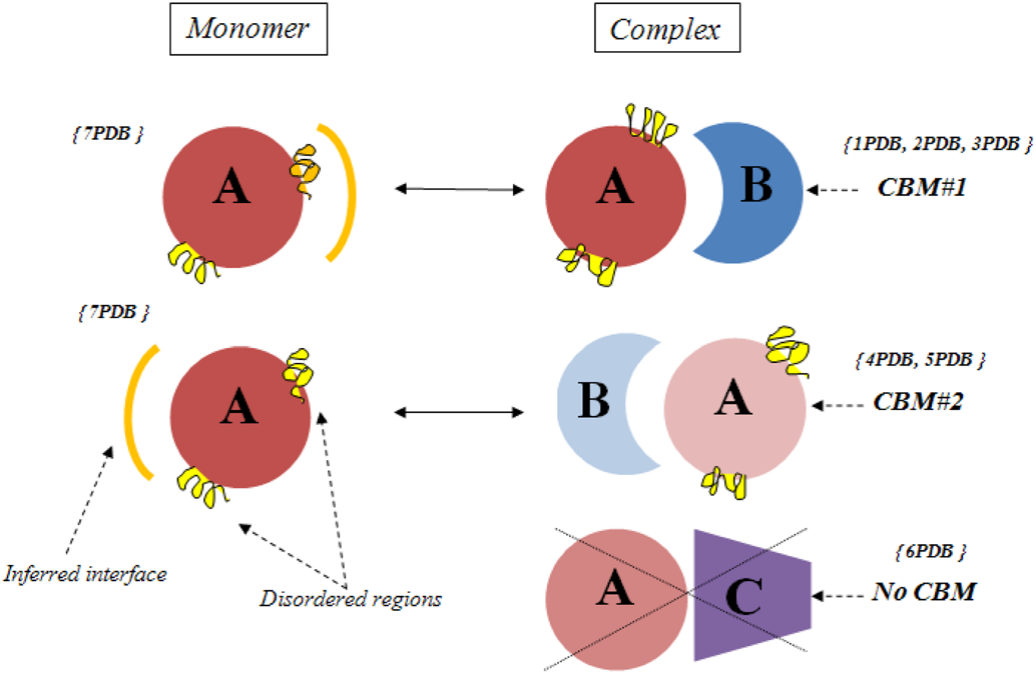
Diagram illustrating the definition of conserved binding modes and construction of the test set. Structures *1PDB* , *2 PDB* and *3PDB* have a recurrent mode of interaction between families *A* and *B*, this constitutes *CBM#1*. Structures *4PDB* and *5PDB* use another binding mode which is also conserved between two different structures and therefore constitutes *CBM#2*. There is only one instance of binding mode between family *A* and *C*, therefore it is not a CBM. Structures of two different representatives of family *A* in complex and monomeric forms are shown. Interface regions mapped from complex to monomer are shown as orange arcs and disordered regions on the inferred interface regions are shown in orange, all other disordered regions are shown in yellow. Fraction disorder in family *A* in a complex state is calculated by averaging over all structures of a given CBM (*1PDB*, *2PDB*, *3PDB* for *CBM#1* and *4PDB*, *5PDB* for *CBM#2*).

Once CDD families are assigned, we identify all interacting chains within a PDB entry. Two chains qualify as interacting if they have at least 5 residue-residue contacts. A contact takes place between a residue from one chain and a residue from the other when the distance between any non-hydrogen atom of one residue is within 6 Å of any non-hydrogen atom of the other residue. The set of residues which make contacts between the chains form the interface. To ensure that interactions are biological and not spurious, such as from crystal packing, we remove interactions that are not confirmed with additional instances of the same family pair interacting in the same orientation, so-called Conserved Binding Modes (CBM) [48]. These CBMs are defined using structural alignments between different structural instances of the same interacting family pair to confirm overlap of at least 50% of interface residue positions (Figure 2). Two definitions of conserved binding modes (CBMs) have been used: in one case confirmation of a binding mode can occur only between different non-redundant structures; in the other case recurrent interactions might occur within one structure. We refer to a dimer of interacting chains with a distinct CBM as a “complex” although it includes only pairwise interactions and several such “complexes” can be found in one PDB entry.

While analyzing disorder in dimer complexes, we also compare their disorder content with the fraction disorder of the protein in a monomeric state (Figure 1). Monomer and complex chains (as defined in PDB) corresponding to the same domain family were aligned to ensure 100% sequence identity in the non-gapped alignment. Their alignment was extended beyond the CDD footprint region as far as possible. In 95% of all cases the alignment was extended to include the entire shorter chain and in 75% of cases the alignment was extended to include both entire chains from monomer and complex structures (within 1–2 residues from both ends). The alignments are more extensive than footprint regions and cover footprint regions plus C- and N- terminal sequence regions which often do not have coordinates. Biological unit assignments were taken from the PDB asymmetric unit (ASU) assignments and from PISA predictions of multimeric states (which are based on calculation of stability of multimeric states inferred from the crystalline state) [52].

We cannot directly investigate the disorder on the interfaces in complexes as complexes are defined through residue contacts so those interface residue coordinates must be present in PDB files (see definitions of disorder below). As shown in Figure 2, disorder on the interfaces can be inferred by exploiting monomeric states of proteins, using their alignment to map the interface region from a complex onto the monomers. Given the overall numbers of disordered and non-disordered residues in the alignment, the number of residues on the mapped interface and the number of disordered residues on the interface, we can estimate the probability of observing a given number (or higher) of disordered residues on the mapped interfaces purely by chance. Using the binomial test we calculated p-values for all complexes with at least five disordered residues in the footprint or aligned regions and at least one disordered residue on the mapped interface (altogether there are 55 complexes for which interface p-values can be calculated).

After excluding those cases where interfaces are entirely outside of the alignment, our data set contained 4,884 dimer complexes and 418 unique monomer structures. Since multiple protein chains can be found in the same PDB entry (on average four chains per PDB entry from our test set) and these chains may belong to the same family, we performed an averaging of all observed quantities over the members of the family and conserved binding modes. Namely, as shown in Figure 2, disorder content observed in family type *X* was averaged over all instances (structures) of family *X* interacting with family type *Y* through a specific CBM. Hereafter we refer to them as “*CBM interactions*” or merely “*interactions*”. Overall, we ended up with 588 CBM interactions (“test588”). To compare disorder content in monomeric and complex states we used the more strict definitions for both binding modes and oligomerization states (see previous section). If we use the more strict CBM definitions and restrict the monomeric states by PISA (those structures which are monomeric in ASU are also predicted to be monomeric by PISA) the set is reduced to 149 interactions (“test149”). Also, for each protein used in our test set we retrieve the Gene Ontology (GO) functional annotations [53]. All structures, protein families, disorder content, GO functional annotations and other relevant information are provided in the Supporting Information.

### Defining disordered regions

Disordered regions were defined as those regions with missing coordinates in X-ray-resolved structures. This is the most direct way to observe intrinsically disordered regions although largely disordered proteins may be underrepresented in PDB because of the difficulties in their crystallization [5]. Disordered regions were also predicted as those with low packing density using the FoldUnfold described previously [31],[32]. Some advantages of the FoldUnfold method are that the program was not trained on the missing coordinates in PDB and that it reports a very high specificity (small number of false positives). Its performance has been shown to be comparable to other disorder prediction methods [31],[54]. (See also Table S2). According to FoldUnfold, an average packing density observed in structures was computed for each of the 20 amino acid residues. These values were considered to be the expected packing density for the same type of residues in a query protein (with or without known structure). Using a sliding window of 11 residues, the center residue of each window is predicted to be disordered if the mean packing density of the window falls below a threshold. We performed disorder predictions for all proteins in our data set.

To differentiate between ordered regions (hinge-like movements or “wobbly” domains, for example) with missing PDB coordinates and true disordered regions, we annotated those regions which are both predicted to be disordered and at the same time have missing coordinates in PDB. They will be referred hereafter as “confirmed disordered regions”. To quantify the disorder content, we calculated the “*fraction disorder*” as a ratio of the number of residues in disordered regions and the number of residues in the footprint or aligned regions. To see all computed values of fraction disorder consult Dataset S1 (missing coordinate definition) and Dataset S2 (confirmed disordered regions).

## Results

### Disorder in protein complexes

**Figure 3:**
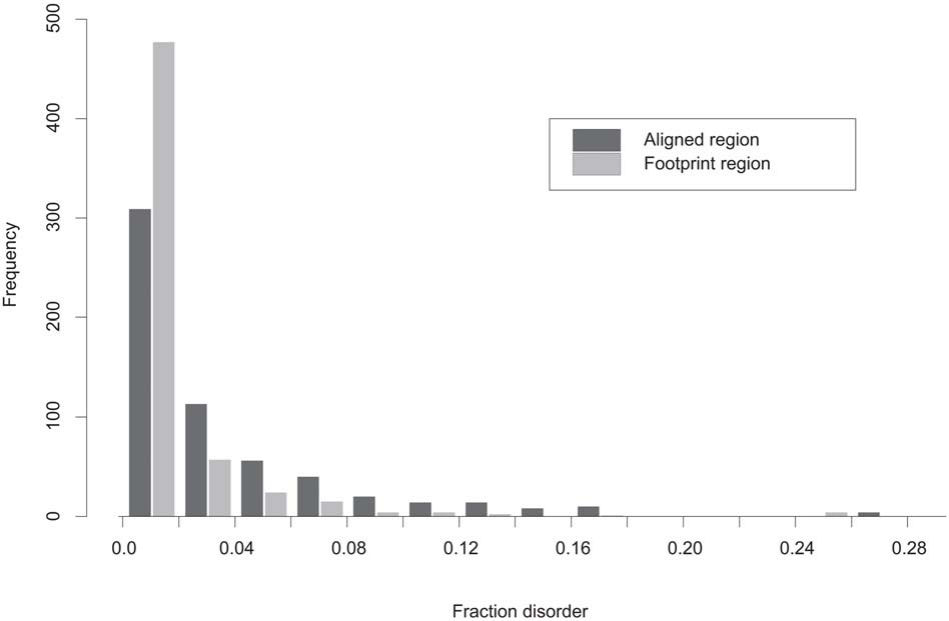
Histogram of fraction disorder in protein complexes (“test588”). Fraction disorder is calculated for aligned (black) and footprint regions (grey). The “footprint region” extends from the first to the last residue in the alignment mapping CDD family to a given chain. “Aligned regions” are more extensive than footprint regions and cover footprint regions plus C and N- terminal protein regions.

Analysis of fraction disorder in different families shows that one quarter of our test complexes do not have any disorder while others can have as much as one third of their residues in the disordered state (Figure 3). The three quarters of complexes with non-zero disorder have on average 4.3% disorder in the aligned regions and about 1.6% in the footprint regions. Confirmed disordered regions have similar disorder content for pairs with non-zero disorder and drops to about 1% if all 588 interactions are included. The reason is that disordered regions with missing coordinates sometimes do not overlap with the predicted disordered regions. There are also families that exhibit rather wide variation in fraction disorder among different members of these families (a ratio of standard deviation over the mean value of fraction disorder is greater than 1); they constitute 13% of all cases.

Table 1 shows several cases of complexes with disorder that were confirmed by experimental studies to be functional. Proteins from these families are found to function in dimer, tetramer and other oligomeric states. Their disordered regions play important roles in regulating the specificity of interactions between the dimer complexes and their interacting partners, in establishing the links between different residues upon allosteric regulation, and possibly in kinetics. In this table we highlight the generality of this phenomenon for many different proteins including enzymes, chaperones and others. As can be seen from this table, all cases (except for the last one) constitute homodimer complexes and, as will be shown in the next section, homodimers have a tendency to contain larger fractions of disordered regions compared to heterodimers. References for Table 1 can be found in Table S1(a).

**Table 1:** Examples of protein complexes with disorder.

Family name, interacting partner, CBM	PDB code	DO, %	Function of disordered region
Chaperone hchA PRK04155 - PRK04155, CBM#83,80	1PV2	8.8 (8.4)	Disorder of loops D2 and D3 leads to the exposure of a hydrophobic patch of dimer interface that helps in binding to client proteins.
Holliday junction resolvases cd00523 – cd00523, CBM#5	1OB8	6.3 (8.8)	Catalytic Ser is located on disordered loop on the junction-binding surface. Accounts for specific binding of four-way DNA junctions.
Pyridoxamine 5′-phosphate oxidase PRK05679-PRK05679, CBM#26	1WV4	25 (28)	Disordered domain can rotate to allow passage of pyridoxal 5′-phosphate.
2-dehydro-3-deoxyphosphooctonate aldolase PRK05198-PRK05198, CBM#202,203,206	1D9E	7.0 (6.5)	Possible role of disorder in homotetrameric enzyme to be involved in synthase kinetic mechanism.
Thymidylate kinase PRK07933 – PRK07933, CBM#1192	1N5K	5.9 (7.2)	Disordered LID region closes on the phosphoryl donor when it binds. It anchors Mg ion, which establishes a link, through Glu166 and Asp9, between the P-loop and the LID region.
Lysin cd00243 - cd00243, CBM#3	2LYN	0.0 (6.9)	Disordered N- and C-termini are involved in the cleft formation which in turn is involved in an initial species-specific binding of the lysin dimer to VERL.
2-methylisocitrate lyase PRK11320 – PRK11320, CBM#72, 77, 74	1S2V	3.6 (2.3)	Disordered loop which is located near dimerization interface serves to gate the PEP mutase active site, converting between an open conformation that allows substrate binding and product release and a closed conformation that separates the reaction site from the solvent during catalysis.
HPr Serine kinase C-terminus, PTS HPr pfam07475 – pfam00381, CBM 9	1KKL	16.3 (4.8)	In complex with serine-phosphorylated Hpr, the disordered loop is a part of interaction interface. The phosphoserine forms an additional residue contact that helps to stabilize the loop.

Columns list names of protein families together with the name of interacting partner, CBM identifier, PDB codes of structural representatives of a complex and a monomer, binomial p-value, percent of disordered residues on a monomer (monomer assignments were taken from ASU and in all but one case were confirmed by PISA) corresponding to the interface region of a complex and description of function of disordered region (references are given in SM). Fraction disorder on interface is averaged over different conserved binding modes of a given family. For references, see Table S1(b).

**Figure 4:**
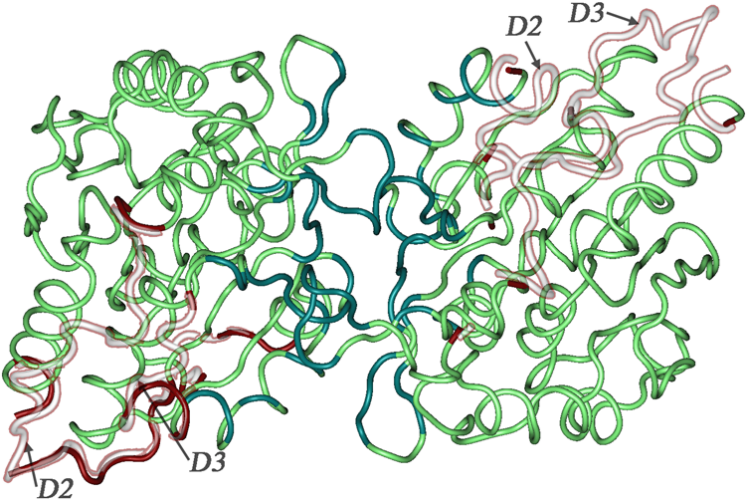
Disordered regions displayed in the homodimeric complex of heat shock protein hsp31 (1PV2). The structured regions of 1PV2 are shown in green with interface residues highlighted in teal. A trace of possible order in the disordered regions was drawn using the 1ONS structure (the same protein in its monomeric state without disorder) which was structurally superimposed on 1PV2 two times, once for each half of the homodimer. Residues capping disordered regions in the homodimer are colored in red and the corresponding ordered residues from the monomer are drawn as silhouettes. A few additional residues of 1ONS in the left monomer are ordered compared to the same residues on the right and therefore are also colored red.

Here we describe in detail one example from the table: a complex of heat shock protein hsp31 which has chaperone activity and functions as a homodimer in solution (1PV2 [55]) (Figure 4). The complex contains four dimers in a triclinic cell exhibiting a conserved symmetrical homodimer binding mode. Structures of the homodimers show significant fraction disorder of about 8–9% in both aligned and footprint regions. Disordered regions D2 and D3 are found at positions 27–49 and 109–115 and part of the first and the entire second region are also predicted to be disordered by the sequence-based method [32]. These regions have particular functional importance as they are located close to the dimer interface and at high temperatures become disordered and expose a large hydrophobic interface area that helps in binding to client proteins [55]. When the temperature decreases, D2 and D3 lock in certain conformations and facilitate the removal of the client protein from the hydrophobic patch.

### Disorder in homo- and hetero-oligomers

We performed an analysis separating all interacting pairs from our test set into homo- (535 complexes) and heterodimers (53 complexes), where both chains in a pair are classified as belonging to the same or different families respectively. Similarly, the prevalence of homodimers over heterodimers in a cell was reported previously [56]. All homodimers were separated into symmetrical and non-symmetrical classes (“isologous” and “heterologous” according to [57]). We define symmetrical homodimers as those that use more than 80% of the same surface in both subunits for binding (316 complexes); all other homodimer arrangements were defined as non-symmetrical (266 complexes). Some homodimer families have structures belonging to both symmetrical and non-symmetrical classes (near the 80% cutoff) but such cases are rare. Eleven families form both homo- and heterodimers. The majority of such cases are examples of larger complexes where the same protein participates in homo- and hetero-interactions within the same complex.

**Figure 5:**
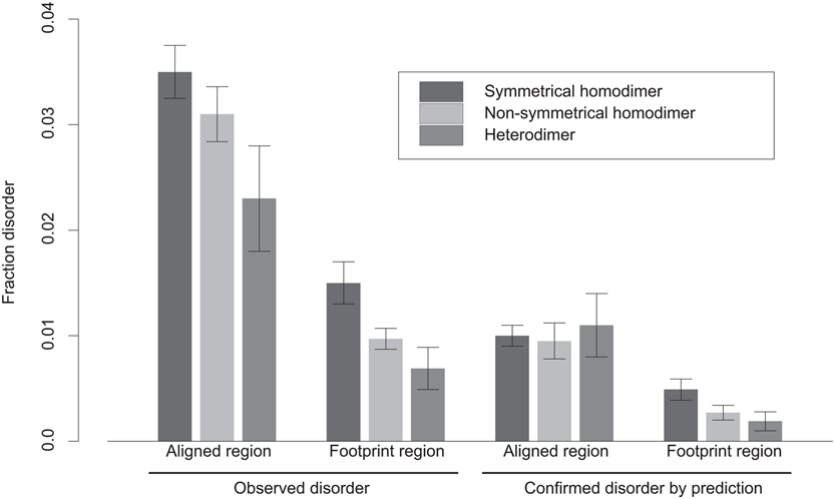
Average fraction disorder together with the standard error is plotted for three categories of oligomers: symmetrical, nonsymmetrical homodimers and heterodimers; for fraction disorder in the aligned and footprint regions. The “footprint region” extends from the first to the last residue in the alignment mapping CDD family to a given chain. “Aligned regions” are more extensive than footprint regions and cover footprint regions plus C and N- terminal protein regions.

Figure 5 shows average fraction disorder in different classes of homo- and heterodimers. As can be seen from this figure, fraction disorder in complexes decreases as the interaction interface deviates more from being a symmetrical homodimer interface. Fraction disorder in heterodimers is almost two times smaller compared to symmetrical homodimers and the difference is statistically significant (p-value<0.001). The observed trend for hetero- and non-symmetrical homo-complexes to contain smaller disordered regions was confirmed by the disorder prediction analysis, although the trend is not as pronounced for predicted disorder in aligned regions. We did not find significant differences in fraction disorder between homo- and heterodimers for proteins that participate in homo- and hetero-interactions within the same complex.

### Inferring disorder-to-order transitions and disorder on binding interfaces

In studying disorder in protein complexes, we can use the monomer states of the proteins as references. First we would like to check whether the disorder-to-order transition may occur upon binding; and second, to analyze if this transition happens on binding interfaces. In this section we compared fraction disorder of proteins in their monomer and complex states. By definition, binding interfaces should involve only residues with coordinates and therefore can introduce bias toward ordered regions in the complexes (complexes with the entire interface disordered are not considered in the analysis). Therefore, for fair comparison between monomers and complexes we subtracted the number of disordered residues in a monomer which are mapped onto interfaces in a complex from the overall number of disordered residues in a monomer.

**Figure 6:**
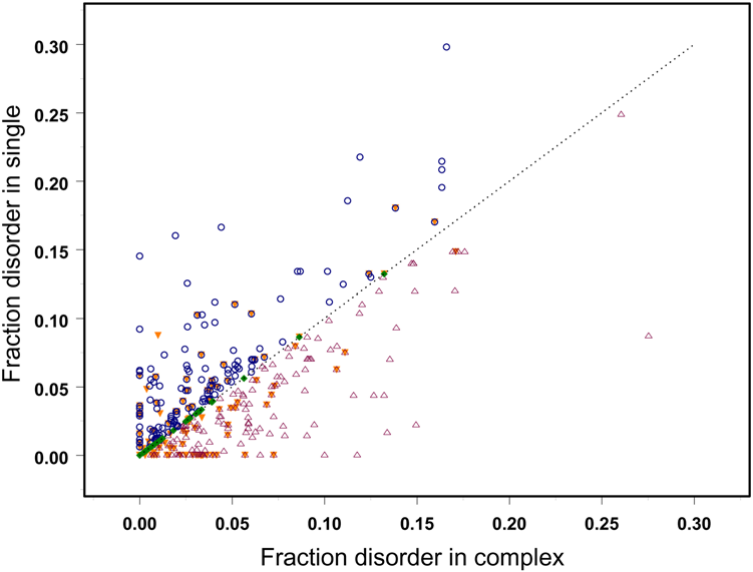
Fraction disorder in the alignment is plotted for monomeric and complex states of each protein averaged over families from “test588”. Those cases with the disorder fraction in a monomer higher, lower or equal to the fraction disorder in a complex are shown with the circles, triangles and diamonds correspondingly. Entries from “test149” are shown as orange upside-down triangles.

Figure 6 shows fraction disorder in aligned regions for monomer and complex structures of the same interaction using the “test588” and “test149” sets. As can be seen from this figure, there exist three types of behavior: cases with higher fraction disorder in a monomer compared to the complex, cases with higher fraction disorder in a complex and, finally, those interactions with no preference towards disordered or ordered states in a monomer or a complex. It should be mentioned that different ways of averaging over structures or using confirmed disorder regions does not change the overall result, namely, that there are three groups and that the sizes of the first and second groups are comparable.

While in the previous section we focused on the disordered regions spanning the whole aligned or footprint regions, here we will focus on disorder in the interface regions. Since the interface in complexes is ordered by definition, we looked at disordered regions in monomers which are aligned to the interface region of the same protein in a complex. The monomer reference state gives us an opportunity to analyze the disorder in the regions of a monomer which form the interface upon binding. We found that the mapped (inferred) interface regions can be up to 50% disordered in a monomer and for 42% of the complexes (23 out of 55 complexes for which p-values can be calculated, see Methods), there is a statistically significant bias toward the disorder on inferred interface regions with p-values of less than 0.05. We observed similar fractions of cases with significant disorder on inferred interfaces if we use confirmed disorder regions (see Methods). Additional restriction of monomeric states by PISA indicates 75% of the cases have significant disorder on interfaces (9 out of 12 complexes from “test149” used for p-value calculation).

**Figure 7:**
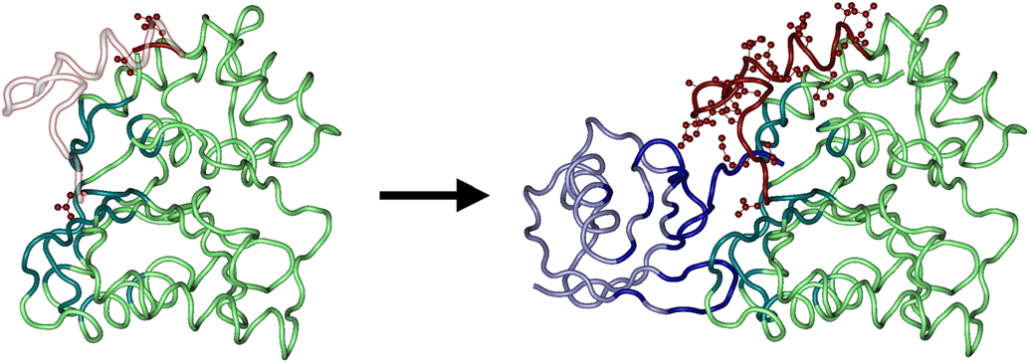
Disorder-to-order transition upon complex formation in ubiquitin *C*-terminal hydrolase. Structures of two forms of the hydrolase are shown to demonstrate the disordered region which becomes ordered upon complex formation. On the left side is the monomeric ubiquitin hydrolase (1UCH) with the residue at either end of the disordered region highlighted in red and shown with sidechains. On the right side is the complex between ubiquitin hydrolase and ubiquitin with the same residues highlighted in red and sidechains drawn. To trace the disordered region, the ordered region on the right has been mapped to the monomeric structure on the left using a superposition between the two structures and is shown as a silhouette.

Several cases with significant disorder on inferred interfaces are listed in Table 2 (and in Table S1(b) to include references). Their disordered regions predicted by FoldUnfold and by five other methods are highlighted in Table S2. Figure 7 shows one example of ubiquitin C-terminal hydrolase in two states: monomeric (1UCH [58]) and in complex (1XD3 [59]) with ubiquitin vinylmethylester, a ubiquitin-based active site-directed probe. Ubiquitin C-terminal hydrolase catalyzes the hydrolysis of the isopeptide linkage between the C-terminal glycine of ubiquitin and a lysine of the target polypeptide. The structure of the free form of this enzyme has 4–6% fraction disorder in footprint and aligned regions compared to only 0–0.9% fraction disorder in the complex with ubiquitin. The disordered region in 1UCH constitutes a 20 residue loop (147–166) which is also predicted to be disordered (region 150–164) by the sequence-based method [32]. This disordered loop is positioned just over the active site cleft and becomes ordered upon binding to ubiquitin vinylmethylester. The interaction interface mapped from complex structure to monomer shows that 30% of the interface is disordered in a monomer (binomial p-value<10^−8^) which points to the coupling between folding and binding. It was suggested earlier that this disordered loop might prevent access to the active site for larger substrates and affect substrate specificity as larger substrates could only be accommodated in the active site by peeling away this loop from the active site cleft [58],[59].

**Table 2:** Examples of protein families with the disorder-to-order transitions on or near interfaces.

Family name, interacting partner, CBM	PDB code	p-value	DO on inferred interface, %	Function of disordered region
Trypsin-like serine protease, BPTI/Kunitz family of serine protease inhibitors smart00020, cd00109, CBM#112, 99	1P2M, 1CHG	1E-8	30.0	Residues which surround active site in chymotripsinogen (monomeric state) become fixed upon activation (in the complex with chymotripsin).
Ephrin receptor ligand binding domain, Ephrin pfam01404, pfam00812, CBM#8, 9	1KGY, 1NUK	1.5E-07	6.8	Unbound ephrin receptor contains partially disordered loops. In the complex (bound to ephrin), these loops are ordered to form the ligand-binding channel.
Malate synthase G cd00728, cd00728, CBM#23	2GQ3, 1N8I	2.5E-09	23.8	One disordered loop region in a monomer forms intermolecular beta sheet with corresponding residues (ordered) on other monomer. The loop ordering suggests an allosteric interaction between the loop and the co-enzyme A binding pocket.
Ubiquitin carboxyl-terminal hydrolase, family 1, Ubiquitin pfam01088, cd01803, CBM#5	1XD3, 1UCH	2.5E-08	30.2	A disordered 20-residue loop is positioned over the active cleft and becomes ordered upon complex formation. It prevents binding the larger substrates and plays role in defining substrate specificity.
Beta-carbonic anhydrase clade C cd03378, cd03378, CBM#47	2A5V, 1YM3	1.3E-06	5.6	Local disorder in a monomer allows active site to be open. In tetramer, the disorder region forms an (ordered) alpha-helix that packs on the other monomer of the essential dimer to create a cavity and restrict access to the active site.
Dihydroneopterin aldolase and 7,8-dihydroneopterin triphosphate epimerase cd00534, cd00534, CBM#497	1NBU, 1Z9W	0.0416	7.7	Enzyme contains a flexible, disordered loop with the catalytic residue Glu22 that hinders active site formation. In allosteric regulation, substrate binding drives conformational changes including ordering of this loop to convert from inactive to active form.
Copropor-phyrinogen III oxidase PRK05330 PRK05330, CBM#8	1TKL, 1TK1	0.0004	30.0	Monomer in an open form has disordered residues on interface which get ordered upon dimer formation.

Columns list names of protein families together with the name of interacting partner, CBM identifier, PDB codes of structural representatives of a complex and a monomer, binomial p-value, percent of disordered residues on a monomer (monomer assignments were taken from ASU and in all but one case were confirmed by PISA) corresponding to the interface region of a complex and description of function of disordered region (references are given in SM). Fraction disorder on interface is averaged over different conserved binding modes of a given family. For references, see Table S1(b).

## Discussion

Our large-scale study of disordered regions in proteins and protein complexes underscores a fascinating diversity among the biological processes that make use of protein disorder. Analysis of GO functional annotations of complexes reveals a variety of categories where intrinsic disorder can play an important functional role, the most frequent of them being nucleic acid binding proteins, enzymes, ATP binding proteins, receptor binding proteins and other ligand binding proteins (see Dataset S3). In addition to well-documented cases of signaling and transcription related proteins, we detect and describe intrinsic disorder in a large variety of enzymes and other proteins. In accordance with the conventional view that folding of disordered regions occurs upon binding to the interaction partners, we find many such cases in our analysis where ordering occurs upon complex formation. Moreover, we investigated the details of protein interaction interfaces and deduced changes occurring on the interfaces in disorder-to-order transitions. We find that in 42–75% of interactions (for which statistical significance could be estimated), there is evidence that disorder-to-order transition occurs on binding interfaces.

Many complexes in our dataset have significant amounts of intrinsic disorder. The role of disordered regions in complexes has been analyzed in several previous studies on smaller test sets [22],[60]. In our study we find as many cases with disorder in complexes as the number of instances of disorder-to-order transition upon binding. This is a rather unusual result as many such cases until recently were largely overlooked. It has been proposed that disordered regions can be energetically beneficial in proteins and their complexes due to a number of reasons: they can provide an increase in backbone conformational entropy upon ligand binding, can accommodate sites for post-translational modifications, and can provide interfaces for binding other partners [6], [22], [60]–[65]. In addition, the formation of complexes of proteins containing functionally important disordered regions can help to increase their stability (entropy-driven complexation, see the last section) and prevent their degradation.

Many proteins perform their functions while interacting with each other in larger complexes. We argue that intrinsic disorder in complexes may play an important functional role in regulating the specificity of interactions between the dimer complexes and their interacting partners, in establishing the links between different residues upon allosteric regulation, and in possibly influencing the kinetics. For example, the mechanisms of regulation of binding specificity through disordered regions in complexes can be as common as for unbound proteins: controlling the exposure of the dimer interface or nearby regions for potential binding targets, or providing specific binding for substrates of certain sizes. The former mechanism has been recently investigated in the stable symmetrical homodimers, UmuD_2_ and UmuD_2_′, which lack secondary structure and might lock the disordered regions in conformations that facilitate further binding of other proteins [66]. In addition, the formalism of flexible folding and mechanism of the “conformational selection” model [19], [67]–[72] can be expanded to include the binding between protein complexes and their interacting partners.

Interestingly, we find that the disorder content in homodimers, especially in symmetrical homodimers, is significantly higher than in heterodimers. Indeed, many soluble and membrane-bound proteins form homo-oligomeric complexes in a cell and oligomerization can generate new binding sites at dimer interfaces to increase specificity and diversity in the formation of complexes. Indeed, intrinsic disorder in homodimers might have more pronounced functional importance compared to the disorder in heterodimeric complexes. Symmetrical arrangements in homodimers might be crucial to keep functional disordered regions close together in space to form joint binding interfaces or to form near-interface regions to regulate the accessibility of the binding partner. Moreover, from the energetic point of view, symmetrical homodimers have an advantage over non-symmetrical arrangements [73],[74]; at the same time, self-interactions between disordered parts in homodimers can be of evolutionary and functional importance [66],[75].

Another explanation comes from thermodynamics considerations. Entropy of complexation gives an important contribution to the complex stability and drives macromolecular complexes to less symmetric states. Any rearrangement of monomers that decrease complex symmetry would therefore result in a more stable complex (see Eq. 20 in [52]). The presence of disordered regions in the symmetrical homodimers will make the protomers asymmetric and change the symmetry number γ from 2 to 1 (two-fold symmetry to asymmetry) and make a favorable contribution to the free energy. At the same time disordered regions should not affect symmetry numbers in cases of heterodimers or non-symmetrical homodimers (they are asymmetric by default) and will not change their stability. Ultimately, the interplay between the binding energy and entropy contributions is important and it is not unrealistic that the entropy-driven disordered complex formation can be realized in some cases.

It is difficult to systematically account for all factors which influence the fraction disorder in proteins. The amount of disorder in crystals depends in general on crystallization conditions and crystal packing parameters. The balance between order and disorder is rather subtle and is difficult to detect but the evidence pointing to the tremendous importance of intrinsic disorder in a large variety of cellular processes is accumulating and merits further study.

## Supporting Information

Table S1 Tables 1 and 2 with references
(0.02 MB PDF)

Table S2 Comparison of different disorder prediction methods for proteins from Table 2
(0.03 MB PDF)

Dataset S1 Fraction disorder for each pair of interacting chains using disorder defined as regions with missing coordinates
(0.03 MB TXT)

Dataset S2 Fraction disorder for each pair of interacting chains using disorder defined as the intersection of regions with missing coordinates and predicted disordered regions
(0.00 MB TXT)

Dataset S3 Functional annotations of complexes
(0.25 MB TXT)
