# Supplementary material for: Intrinsic Disorder in Protein Interactions: Insights From a Comprehensive Structural Analysis
Source: PLoS Comput Biol. 2009 Mar 13;5(3):e1000316. doi: 10.1371/journal.pcbi.1000316 (PMC2646137; doi:10.1371/journal.pcbi.1000316)
Supplement: Table S1 — Tables 1 and 2 with references (0.02 MB PDF) [file pcbi.1000316.s001.pdf]

Table S1. Tables 1 and 2 with references.

(a) **Examples of protein complexes with disorder**. (Same as Table 1, with references.) Columns list names of protein families together with the name of interacting partner and their respective domain accession codes, and CBM identifier(s); PDB code of structural representatives of a complex; the fraction of footprint disorder, DO (fraction disorder in aligned regions is shown in brackets); and description of function of disordered region (according to the paper reporting a given structure). Fraction disorder listed in this table is averaged over different conserved binding modes of a given family. Oligomeric state assignments for all cases were confirmed by PISA as being dimers and tetramers.

Domain name, interacting partner, CBM	PDB code	DO (%)	Function of disordered region
Chaperone hchA, PRK04155-PRK04155, CBM#83,80	1PV2	8.8 (8.4)	Disorder of loops D2 and D3 leads to the exposure of a hydrophobic patch of dimer interface that helps in binding to client proteins [1].
Holliday junction resolvases, cd00523-cd00523, CBM#5	1OB8	6.3 (8.8)	Catalytic Ser is located on disordered loop on the junction-binding surface. Accounts for specific binding of four-way DNA junctions [2].
Pyridoxamine 5'-phosphate oxidase, PRK05679-PRK05679, CBM#26	1WV4	25 (28)	Disordered domain can rotate to allow passage of pyridoxal 5'-phosphate [3].
2-dehydro-3-deoxyphosphooctonate aldolase, PRK05198-PRK05198, CBM#202,203,206	1D9E	7.0 (6.5)	Possible role of disorder in homotetrameric enzyme to be involved in synthase kinetic mechanism [4].
Thymidylate kinase, PRK07933-PRK07933, CBM#1192	1N5K	5.9 (7.2)	Disordered LID region closes on the phosphoryl donor when it binds. It anchors Mg ion, which establishes a link, through Glu166 and Asp9, between the P-loop and the LID region [5].
Lysin, cd00243-cd00243, CBM#3	2LYN	0.0 (6.9)	Disordered N- and C-termini are involved in the cleft formation which in turn is involved in an initial species-specific binding of the lysin dimer to VERL [6].
2-methylisocitrate lyase, PRK11320-PRK11320, CBM#72, 77, 74	1S2V	3.6 (2.3)	Disordered loop which is located near dimerization interface serves to gate the PEP mutase active site, converting between an open conformation that allows substrate binding and product release and a closed conformation that separates the reaction site from the solvent during catalysis [7].
HPr Serine kinase C-terminus, PTS HPr, pfam07475-pfam00381, CBM#9	1KKL	16.3 (4.8)	In complex with serine-phosphorylated Hpr, the disordered loop is a part of interaction interface. The phosphoserine forms an additional residue contact that helps to stabilize the loop [8].

(b) Examples of protein families with the disorder to-order transitions on interfaces. (Same as Table 2, with references.) Columns list names of protein families together with the name of interacting partner and their respective domain accession codes, and CBM identifier(s); PDB codes of structural representatives of a complex and a monomer; binomial p-value; percent of disordered residues on a monomer (monomer assignments were taken from PDB and in all but one case were confirmed by PISA) corresponding to the interface region of a complex; and description of function of disordered region (according to the paper reporting a given structure). Fraction disorder on interface is averaged over different conserved binding modes of a given family.

Domain name, interacting partner, CBM	PDB code	p-value	DO on interface (%)	Function of disordered region
Trypsin-like serine protease, BPTI/Kunitz serine protease inhibitors, smart00020-cd00109, CBM#112, 99	1P2M, 1CHG	1E-8	30.0	Residues which surround active site in chymotripsinogen (monomeric state) become fixed upon activation (in the complex with chymotripsin) [9,10,11,12].
Ephrin receptor ligand binding domain, Ephrin, pfam01404-pfam00812, CBM#9	1KGY, 1NUK	1.5E-07	6.8	Unbound ephrin receptor contains partially disordered loops. In the complex (bound to ephrin), these loops are ordered to form the ligand-binding channel [13,14].
sMalate synthase G, cd00728-cd00728, CBM#23	2GQ3, 1N8I	2.5E-09	23.8	One disordered loop region in a monomer forms intermolecular beta sheet with corresponding residues (ordered) on other monomer. The loop ordering suggests an allosteric interaction between the loop and the co-enzyme A binding pocket [15,16].
Ubiquitin carboxyl-terminal hydrolase, family 1, Ubiquitin, pfam01088-cd01803, CBM#5	1XD3, 1UCH	2.5E-08	30.2	A disordered 20-residue loop is positioned over the active cleft and becomes ordered upon complex formation. It prevents binding the larger substrates and plays role in defining substrate specificity [17,18].
Beta-carbonic anhydrase clade C, cd03378-cd03378, CBM#47	2A5V, 1YM3	1.3E-06	5.6	Local disorder in a monomer allows active site to be open. In tetramer, the disorder region forms an (ordered) alpha-helix that packs on the other monomer of the essential dimer to create a cavity and restrict access to the active site [19,20].
Dihydroneopterin aldolase and 7,8-dihydroneopterin triphosphate epimerase, cd00534-cd00534, CBM#497	1NBU, 1Z9W	0.0416	7.7	Enzyme contains a flexible, disordered loop with the catalytic residue Glu22 that hinders active site formation. In allosteric regulation, substrate binding drives conformational changes including ordering of this loop to convert from inactive to active form [21].
Copropor-phyrinogen III oxidase, PRK05330-PRK05330, CBM#8	1TKL, 1TK1	0.0004	30.0	Monomer in an open form has disordered residues on interface which get ordered upon dimer formation [22].

References

1. Quigley PM, Korotkov K, Baneyx F, Hol WG (2004) A new native EcHsp31 structure suggests a key role of structural flexibility for chaperone function. Protein Sci 13: 269-277.
2. Middleton CL, Parker JL, Richard DJ, White MF, Bond CS (2004) Substrate recognition and catalysis by the Holliday junction resolving enzyme Hje. Nucleic Acids Res 32: 5442-5451.
3. Safo MK, Musayev FN, Schirch V (2005) Structure of Escherichia coli pyridoxine 5'-phosphate oxidase in a tetragonal crystal form: insights into the mechanistic pathway of the enzyme. Acta Crystallogr D Biol Crystallogr 61: 599-604.
4. Kohen A, Jakob A, Baasov T (1992) Mechanistic studies of 3-deoxy-D-manno-2-octulosonate-8-phosphate synthase from Escherichia coli. Eur J Biochem 208: 443-449.
5. Li de la Sierra I, Munier-Lehmann H, Gilles AM, Barzu O, Delarue M (2001) X-ray structure of TMP kinase from Mycobacterium tuberculosis complexed with TMP at 1.95 A resolution. J Mol Biol 311: 87-100.
6. Kresge N, Vacquier VD, Stout CD (2000) 1.35 and 2.07 A resolution structures of the red abalone sperm lysin monomer and dimer reveal features involved in receptor binding. Acta Crystallogr D Biol Crystallogr 56: 34-41.
7. Liu S, Lu Z, Han Y, Jia Y, Howard A, et al. (2004) Conformational flexibility of PEP mutase. Biochemistry 43: 4447-4453.
8. Fieulaine S, Morera S, Poncet S, Mijakovic I, Galinier A, et al. (2002) X-ray structure of a bifunctional protein kinase in complex with its protein substrate HPr. Proc Natl Acad Sci U S A 99: 13437-13441.
9. Helland R, Czapinska H, Leiros I, Olufsen M, Otlewski J, et al. (2003) Structural consequences of accommodation of four non-cognate amino acid residues in the S1 pocket of bovine trypsin and chymotrypsin. J Mol Biol 333: 845-861.
10. Czapinska H, Helland R, Smalas AO, Otlewski J (2004) Crystal structures of five bovine chymotrypsin complexes with P1 BPTI variants. J Mol Biol 344: 1005-1020.
11. Scheidig AJ, Hynes TR, Pelletier LA, Wells JA, Kossiakoff AA (1997) Crystal structures of bovine chymotrypsin and trypsin complexed to the inhibitor domain of Alzheimer's amyloid beta-protein precursor (APPI) and basic pancreatic trypsin inhibitor (BPTI): engineering of inhibitors with altered specificities. Protein Sci 6: 1806-1824.
12. Freer ST, Kraut J, Robertus JD, Wright HT, Xuong NH (1970) Chymotrypsinogen: 2.5-angstrom crystal structure, comparison with alpha-chymotrypsin, and implications for zymogen activation. Biochemistry 9: 1997-2009.
13. Himanen JP, Rajashankar KR, Lackmann M, Cowan CA, Henkemeyer M, et al. (2001) Crystal structure of an Eph receptor-ephrin complex. Nature 414: 933-938.
14. Himanen JP, Henkemeyer M, Nikolov DB (1998) Crystal structure of the ligand-binding domain of the receptor tyrosine kinase EphB2. Nature 396: 486-491.
15. Anstrom DM, Remington SJ (2006) The product complex of M. tuberculosis malate synthase revisited. Protein Sci 15: 2002-2007.
16. Smith CV, Huang CC, Miczak A, Russell DG, Sacchettini JC, et al. (2003) Biochemical and structural studies of malate synthase from Mycobacterium tuberculosis. J Biol Chem 278: 1735-1743.
17. Johnston SC, Larsen CN, Cook WJ, Wilkinson KD, Hill CP (1997) Crystal structure of a deubiquitinating enzyme (human UCH-L3) at 1.8 A resolution. Embo J 16: 3787-3796.
18. Misaghi S, Galardy PJ, Meester WJ, Ovaa H, Ploegh HL, et al. (2005) Structure of the ubiquitin hydrolase UCH-L3 complexed with a suicide substrate. J Biol Chem 280: 1512-1520.

19. Covarrubias AS, Bergfors T, Jones TA, Hogbom M (2006) Structural mechanics of the pH-dependent activity of beta-carbonic anhydrase from Mycobacterium tuberculosis. J Biol Chem 281: 4993-4999.
20. Suarez Covarrubias A, Larsson AM, Hogbom M, Lindberg J, Bergfors T, et al. (2005) Structure and function of carbonic anhydrases from Mycobacterium tuberculosis. J Biol Chem 280: 18782-18789.
21. Goulding CW, Apostol MI, Sawaya MR, Phillips M, Parseghian A, et al. (2005) Regulation by oligomerization in a mycobacterial folate biosynthetic enzyme. J Mol Biol 349: 61-72.
22. Phillips JD, Whitby FG, Warby CA, Labbe P, Yang C, et al. (2004) Crystal structure of the oxygen-dependant coproporphyrinogen oxidase (Hem13p) of Saccharomyces cerevisiae. J Biol Chem 279: 38960-38968.
