# Supplementary material for: Intrinsic Disorder in Protein Interactions: Insights From a Comprehensive Structural Analysis
Source: PLoS Comput Biol. 2009 Mar 13;5(3):e1000316. doi: 10.1371/journal.pcbi.1000316 (PMC2646137; doi:10.1371/journal.pcbi.1000316)
Supplement: Table S2 — Comparison of different disorder prediction methods for proteins from Table 2 (0.03 MB PDF) [file pcbi.1000316.s002.pdf]

Table S2.

(a) Comparison of different disorder prediction methods for proteins from Table 2. Each protein is referenced below by the PDB code of the monomer. Its family and disordered regions with respect to missing X-ray coordinates in the monomer structure and six prediction methods are listed. Table 2 contains additional information including protein family, interacting partner, and function.

Protein (family)	Missing coordinates	Predicted disordered regions					
		VSL2 [1]	VL3H* [2]	FoldUnfold [3]	IUPred* [4]	RONN [5]	DISOPRED [6]
1chg smart00020	70-77, 143-153	1-4, **70-82**, **151-155**, 189-194, 219-224, 242-245	87-101	**71-82**	1-6, 242-245	4-9, **70-81,** 113-137, **147-165**	1, 5-7, 244, 245
1nuk pfam01404	32-34, 133-136, 177-183	1-9, 24-28, 81-84, **181-183**	1-19	-	1-12, **183**	82-84	**183**
1n8i cd00728	1,71-76, 302-311, 381-385, 674-677, 728-741	**1-6**, 150-160, 182-188, 466-481, **706-741**	194-198, **709-741**	148-163, 217-227, **722-741**	103-105, 153-159, 471-477, **718-741**	146-163, 208-220, 468-484, 507-508, 536-557, **705-741**	157-158, **721-741**
1uch pfam01088	1-4, 147-166	**1-6**, 67-80, 108-139, **148-162**, 192-200, 227-230	**1-3**, 112-149, 199-205, 222-230	65-80, **150-164,** 189-202	67-70, **148-159**	68-82, 112-130, **145-164**, 190-194	65-78, **150-168**
1ym3 cd03378	1-11 (His-tag)** 40-49 215	**1-44**, 156-162, 181-182, **210-215**	**1-35**, 155-170	**31-50**	**1-17, 27-44,**	**1-49**	**1-15**
1z9w cd00534	1, 15-25, 121-133	**1-6**, **19-20**, 45-47, **128-133**	3-6, 103-104, **129-133**	-	-	-	-
1tk1 PRK05330	1-2, 37-45, 87-101, 187-201	**1-8**, **41-50, 80-96**, 120-122, 170-175, 216, 244-260	1-14, 249-260	**40-53, 87-100**	**1-14**, **37-41**, 43-56 **81-94**	**1**, **35-47, 81-94**, 249-260	**1-4**, **79-92**

*For these methods, those residues which are predicted as having >50% probability of being disordered, were considered as disordered.

**His-tag was not considered in evaluations.

(b) Quality of predictions of disordered (and ordered) residues by different methods for proteins from Table 2.

	VSL2	VL3H	FoldUnfold	IUPred	RONN	DISOPRED
TP	83	30	66	53	78	43
FP	262	179	86	97	236	45
TN	1560	1643	1736	1725	1586	1777
FN	94	147	111	124	99	134
$Sensitivity$	0.47	0.17	0.37	0.30	0.44	0.24
$Specificity$	0.86	0.90	0.95	0.95	0.87	0.98
$1-Specificity$	0.14	0.10	0.05	0.05	0.13	0.02
$S\_product$	0.40	0.15	0.36	0.28	0.38	0.24

TP (true positives) is number of residues correctly predicted as being disordered;
TN (true negatives) is number of residues correctly predicted as being ordered;
FP (false positives) is number of ordered residues predicted as disordered;
FN (false negatives) is number of disordered residues predicted as ordered;

$$Sensitivity = \frac{TP}{TP+FN};$$

$$Specificity = \frac{FP}{FP+TN};$$

$$S\_product = Sensitivity \times Specificity.$$

References:

1. Obradovic Z, Peng K, Vucetic S, Radivojac P, Dunker AK (2005) Exploiting heterogeneous sequence properties improves prediction of protein disorder. Proteins 61 Suppl 7: 176-82.
2. Obradovic Z, Peng K, Vucetic S, Radivojac P, Brown CJ, Dunker AK (2003) Predicting intrinsic disorder from amino acid sequence. Proteins 53 Suppl 6: 566-72
3. Galzitskaya OV, Garbuzynskiy SO, Lobanov MY (2006) FoldUnfold: web server for the prediction of disordered regions in protein chain. Bioinformatics 22: 2948-2949.
4. Dosztanyi Z, Csizmok V, Tompa P, Simon I (2005) IUPred: web server for the prediction of intrinsically unstructured regions of proteins based on estimated energy content. Bioinformatics 21: 3433-3434.
5. Yang ZR, Thomson R, McNeil P, Esnouf RM (2005) RONN: the bio-basis function neural network technique applied to the detection of natively disordered regions in proteins. Bioinformatics 21: 3369-3376.
6. Ward JJ, Sodhi JS, McGuffin LJ, Buxton BF, Jones DT (2004) Prediction and functional analysis of native disorder in proteins from the three kingdoms of life. J Mol Biol 337: 635-645.
